# Prevalence and factors associated with metabolic syndrome in university students and academic staff in Bangladesh

**DOI:** 10.1038/s41598-023-46943-x

**Published:** 2023-11-14

**Authors:** Nurshad Ali, Mitu Samadder, Jahid Hasan Shourove, Abu Taher, Farjana Islam

**Affiliations:** 1 Department of Biochemistry and Molecular Biology, Shahjalal University of Science and Technology, Sylhet, 3114 Bangladesh; 2 Department of Food Engineering and Tea Technology, Shahjalal University of Science and Technology, Sylhet, 3114 Bangladesh

**Keywords:** Metabolic disorders, Epidemiology

## Abstract

Metabolic syndrome (MetS) is a group of medical conditions that increase the risk of cardiovascular disease, stroke, and type 2 diabetes. While there are numerous studies on the prevalence of MetS in the general adult population worldwide, limited information exists regarding its prevalence among university students and academic staff. This study aimed to determine the prevalence of MetS and associated risk factors among Bangladesh university students and academic staff. For this cross-sectional study, 583 participants were randomly selected from university students (n = 281) and academic staff (n = 302) in Bangladesh. The participants' fasting blood samples were collected, and their serum lipid profile levels, fasting blood glucose, and other parameters were measured using standard methods. MetS was defined according to the NCEP-ATP III model guidelines. Additionally, a questionnaire was administered to the participants to gather information on socio-demographics, lifestyle risk behaviours, and personal medical history. Multivariate logistic regression models were used to determine the risk factors associated with MetS. Overall, the prevalence of MetS was 27.7% in students and 47.7% in staff. There was a significant difference (p < 0.01) in MetS prevalence between male students (34.8%) and female students (17.2%). In contrast, it was comparatively higher in female staff (52.3%) than in male staff (45.8%), although the difference was not statistically significant. The prevalence of MetS and its components increased with age in student and staff groups. The most common component of MetS was low levels of HDL-C, which affected 78% and 81.4% of the students and staff, respectively. Logistic regression modelling showed that increased age, BMI, hypertension, dyslipidemia, low physical activity, and smoking were significantly associated with MetS in students (at least p < 0.05 for all cases). On the other hand, increased age and BMI, hypertension, and dyslipidemia were significantly associated with MetS in academic staff (at least p < 0.05 for all cases). In conclusion, this study indicates a high prevalence of MetS in university students and staff in Bangladesh. Age, BMI, hypertension and dyslipidemia were independently associated with the risk of MetS in both groups. The findings emphasize the importance of interventions for students and staff in academic settings in Bangladesh. It is crucial to implement health promotion activities such as healthy diet and exercise programs more rigorously. Further research with more representative samples is needed to get more clear insights into MetS prevalence in this particular population subgroup for targeted interventions.

## Introduction

Non-communicable diseases (NCDs) are a major concern for public health in developing countries and are responsible for the highest number of deaths and disease burden worldwide. It has been estimated that 74% of all global deaths per year are caused by NCDs, with 77% of these occurring in low- and middle-income countries^1^. NCDs have placed a significant burden on health systems and economies globally. Metabolic Syndrome (MetS) is a condition that raises the likelihood of developing chronic NCDs, particularly cardiovascular disease^2^. MetS is a group of at least three of five metabolic abnormalities occurring together^3^. These include high blood sugar, high blood pressure, elevated levels of triglycerides, low levels of high-density lipoprotein cholesterol, and an increase in waist circumference^3^. MetS is primarily caused by a combination of overeating and a lack of physical activity^4^. This leads to the accumulation of excess body fat, eventually resulting in other metabolic issues^4^. MetS is a contributing factor in the development of various health issues such as diabetes, stroke, advanced colorectal polyps, and cardiovascular diseases^3,5^.

The global prevalence of MetS varied from 12.5 to 31.4% based on its definition^6^. A review conducted in South Asia reported that the prevalence of MetS was 14.0% (WHO), 26.1% (ATP III), 29.8% (IDF), and 32.5% (modified ATP III)^7^. Bangladesh, a developing country in South Asia, is particularly at risk for NCDs^8^. Non-communicable chronic diseases and associated mortality have significantly increased in Bangladesh due to rapid urbanization and changes in food habits, such as increased access to processed food, irregular meal times, and less physical activity^9,10^. A systematic review and meta-analysis reported the prevalence of metabolic syndrome as 37.0% in the Bangladeshi population^11^. The high prevalence of MetS is a pressing public health issue that requires exploration of potential risk factors.

Over the past few years, there has been a noticeable increase in obesity among university students^12,13^. Studies have shown that regardless of age or education, certain lifestyle factors can contribute to the development of MetS, which can lead to chronic health complications^14^. Teaching is an important profession that does not require strenuous physical activity but involves stress. High levels of sedentary behaviour and low physical activity can increase the risk of MetS among teaching staff. It was observed in many studies that metabolic syndrome and cardiovascular diseases are rising in the sedentary population^15,16^. Although few studies have reported the prevalence of MetS in general adults in Bangladesh^11,17^, there is a lack of or limited data on the prevalence of MetS in the university context in Bangladesh. Moreover, it is important to know the prevalence of MetS and associated risk factors among university students and academic staff in Bangladesh to develop effective measures and strategies for identification and management from a public health perspective. This study was conducted to estimate the prevalence of MetS and identify associated risk factors among university students and academic staff in Bangladesh.

## Methods

### Study area and study participants

Between February 2019 and January 2020, this cross-sectional study was conducted at the Biochemistry and Molecular Biology department of Shahjalal University of Science and Technology (SUST), Sylhet 3114, Bangladesh. Data and samples were collected from the university campuses of SUST and another university in the Dhaka district. In total, 583 participants (281 students and 302 academic staff) were randomly selected with at least one year of studying or working experience for students and staff, respectively. Students were enrolled through classroom announcements and word-of-mouth, whereas academic staff were enrolled through email contact and word-of-mouth. We invited around 800 participants, and 583 responded (73%) to participate in the study. We invited approximately 800 participants, out of which 583 participants (73%) expressed their willingness to participate in the study. A simple random sampling technique was used to determine the study population. The sample size calculation was done by PASS version 15.0, where 490 participants (295 males and 195 females) were needed to achieve 90% statistical power. The inclusion criteria were both sexes and at least 18 years old. Exclusion criteria included physical dysfunction, infectious disease, liver and kidney diseases, pregnancy and nursing mothers, incomplete questionnaires or missing blood samples. The Biochemistry and Molecular Biology department, School of Life Sciences, SUST, Ethics Review Committee approved the study protocol (ID 02/BMB/2019). Prior to the start of the study, all participants provided written consent after being fully informed about the details of the study. Before study commencement, all study subjects provided written informed consent. The study's procedures were conducted in adherence to the regulations and guidelines set by the institution.

### Data collection

To gather information about participants' physical characteristics, lifestyle, and demographics, we utilized a structured questionnaire (previously described in^18–27^). Trained researchers took measurements of body height, weight, waist and hip circumference (WC and HC), and calculated the Body Mass Index (BMI) accordingly. Before blood pressure readings were taken, participants were instructed to rest for 10 min. Three consecutive readings were then taken 5 min apart. The average of the second and third readings were used to determine systolic and diastolic blood pressures (SBP and DBP, respectively). An automated blood pressure measuring device (Omron M10, Omron Corporation, Tokyo, Japan) was used for blood pressure measurement.

### Sample collection and laboratory analysis

The participants' venous blood samples were collected in the morning after they had fasted overnight. The serum was extracted from the samples through centrifugation and stored at − 80 °C until the analysis of markers. Enzymatic colourimetric methods were utilized to analyze the levels of serum glucose, creatinine, total cholesterol (TC), triglycerides (TG), low-density lipoprotein (LDL-C), and high-density lipoprotein (HDL-C). All biochemical markers were measured using a semi-automated bioanalyzer (Humalyzer 3000, USA). The biochemical measurements were conducted in a single laboratory, with consistent methods employed throughout the assay period.

### Definition

To determine if someone has metabolic syndrome (MetS), the National Cholesterol Education Program (NCEP)-Adult Treatment Panel III (NCEP-ATP III) criteria were used^28^. This required at least three of the following components: a waist circumference of 90 cm or more in men or 80 cm or more in women; triglyceride (TG) levels of 150 mg/dL or more; high-density lipoprotein cholesterol (HDL-C) levels of less than 40 mg/dL in men, or less than 50 mg/dL in women; blood pressure readings of 130/85 mmHg or more, or taking antihypertensive medication; fasting blood glucose (FBG) levels of 110 mg/dL or more, or taking antidiabetic medications. Dyslipidemia was defined as having at least one of the following: TC ≥ 200 mg/dL; TG: ≥ 150 mg/dL; LDL-C ≥ 130 mg/dL and HDL-C < 40 mg/dL^28^. Physical activity was determined by the Global Physical Activity Questionnaire (GPAQ) proposed by the World Health Organization (WHO)^29^ and is split into three levels: low or sedentary, moderate, and adequate or vigorous^30^.

### Statistical analyses

The data analyses were conducted with SPSS Version 25.0, produced by IBM in Chicago, IL, USA. The results were presented as means, frequencies, and percentages. To compare the means between two groups an independent sample *t*-test was applied, while the Chi-square test was used to compare categorical variables. Multivariate logistic regression was also performed to determine the factors that are independently associated with MetS. MetS was the dependent variable in the regression models, while some baseline parameters, the anthro-demographics and behavioural factors were considered independent variables. All p-values were two-sided, and a p-value of less than 0.05 was considered statistically significant.

## Results

### Participant’s baseline characteristics

The participants' general characteristics are outlined in Table 1. There were 384 male and 199 female participants, making a total of 583. The students' average age was 21.8 ± 2 years, whereas the staff's average age was 40.5 ± 10 years, and there was a significant difference between the male and female groups (p < 0.001). Among the students, only WC and DBP showed a significant difference between the sexes (p < 0.05 for both). Among the staff, males had higher WC, SBP, and DBP averages than females (p < 0.01 for all). According to blood pressure and FBG levels, 11.5% and 1.3% of the students were hypertensive and diabetic, respectively. Among the academic staff, 27% and 7.6% were hypertensive and diabetic, respectively. Regarding biochemical markers, male students had a significantly higher mean TG level, while female students had a higher mean HDL-C level (p < 0.001). Male staff had higher average TC and LDL-C levels, and female staff had slightly higher mean TG and HDL-C levels. Around 81% of the students and 79.4% of the staff engaged in medium/adequate physical activity. Smoking was reported by 16.2% of the students and 11% of the staff.

**Table 1:** Characteristics of the study participants.

Variables	Students				Academic staff			
	Total	Male	Female	P-value	Total	Male	Female	P-value
N	281	168	113		302	216	86	
Age (years)	21.8 ± 2.0	22.2 ± 2.0	21.2 ± 2.0	0.000	40.5 ± 10.0	41.8 ± 10.0	37.3 ± 8.0	0.000
BMI (kg/m^2^)	22.6 ± 4.4	22.9 ± 3.5	22.1 ± 5.6	0.214	25.7 ± 3.12	25.26 ± 2.6	26.8 ± 3.8	0.000
WC (cm)	79.9 ± 8.6	80.8 ± 8.7	77.7 ± 8.1	0.047	86.6 ± 8.1	88.3 ± 6.7	84.4 ± 9.4	0.005
SBP (mmHg)	120.4 ± 66.8	121.1 ± 11.9	119.2 ± 104.9	0.855	120.9 ± 13.4	123.3 ± 13.1	115.2 ± 12.4	0.000
DBP (mmHg)	74.0 ± 9.2	76.3 ± 8.5	70.5 ± 9.1	0.000	82.1 ± 10.2	83.9 ± 9.6	77.7 ± 10.4	0.000
FBG (mg/dL)	78.1 ± 19.8	79.2 ± 21.6	75.6 ± 19.8	0.105	100.7 ± 41.4	99.2 ± 34.2	104.4 ± 54.0	0.292
TG (mg/dL)	146.6 ± 94.9	166.2 ± 102.3	114.1 ± 70.7	0.000	164.4 ± 81.2	159.4 ± 83.6	176.5 ± 74.0	0.119
TC (mg/dL)	161.3 ± 52.5	166.3 ± 54.2	152.8 ± 48.9	0.069	167.4 ± 50.2	169.6 ± 51.1	161.9 ± 47.7	0.204
LDL-C (mg/dL)	100.2 ± 46.3	104.7 ± 47.4	92.8 ± 43.7	0.072	101.3 ± 48.4	105.2 ± 48.4	91.6 ± 47.3	0.027
HDL-C (mg/dL)	35.9 ± 15.0	33.4 ± 13.42	40.1 ± 16.6	0.003	34.2 ± 10.8	34.0 ± 10.4	34.6 ± 12.0	0.685
Hypertensive (%)	11.5	16.9	3.3	0.002	27.0	30.8	17.4	0.018
Diabetic (%)	1.3	0.7	2.2	0.348	7.6	7.9	7.0	0.792
Hypertensive-diabetic	2.6	2.2	3.3	0.616	14.3	14.0	15.1	0.806
Physical activity (%)								
Low	18.8	13.0	27.6	0.126	20.6	19.3	23.2	0.482
Medium/adequate	81.2	87.0	72.4		79.4	80.7	76.8	
Smoking (%)								
No	83.8	77.4	100.0	0.000	89.0	84.7	100	0.000
Yes	16.2	22.6	0.0		11.0	15.3	0	

Data are presented as mean ± SD or %. Hypertensive: participants who only hypertensive without diabetes, Diabetic: participants who were only diabetic without hypertension, hypertensive-diabetic: participants who were both hypertensive and diabetic. *BMI* body mass index, *WC* waist circumference, *SBP* systolic blood pressure, *DBP* diastolic blood pressure, *TG* triglyceride, *TC* total cholesterol, *LDL-C* low density lipoprotein cholesterol, *HDL-C* high density lipoprotein cholesterol. P-values are derived from independent sample *t*-test for continuous variables and Chi-square test for categorical variables.

### Prevalence of MetS by gender

**Figure 1:**
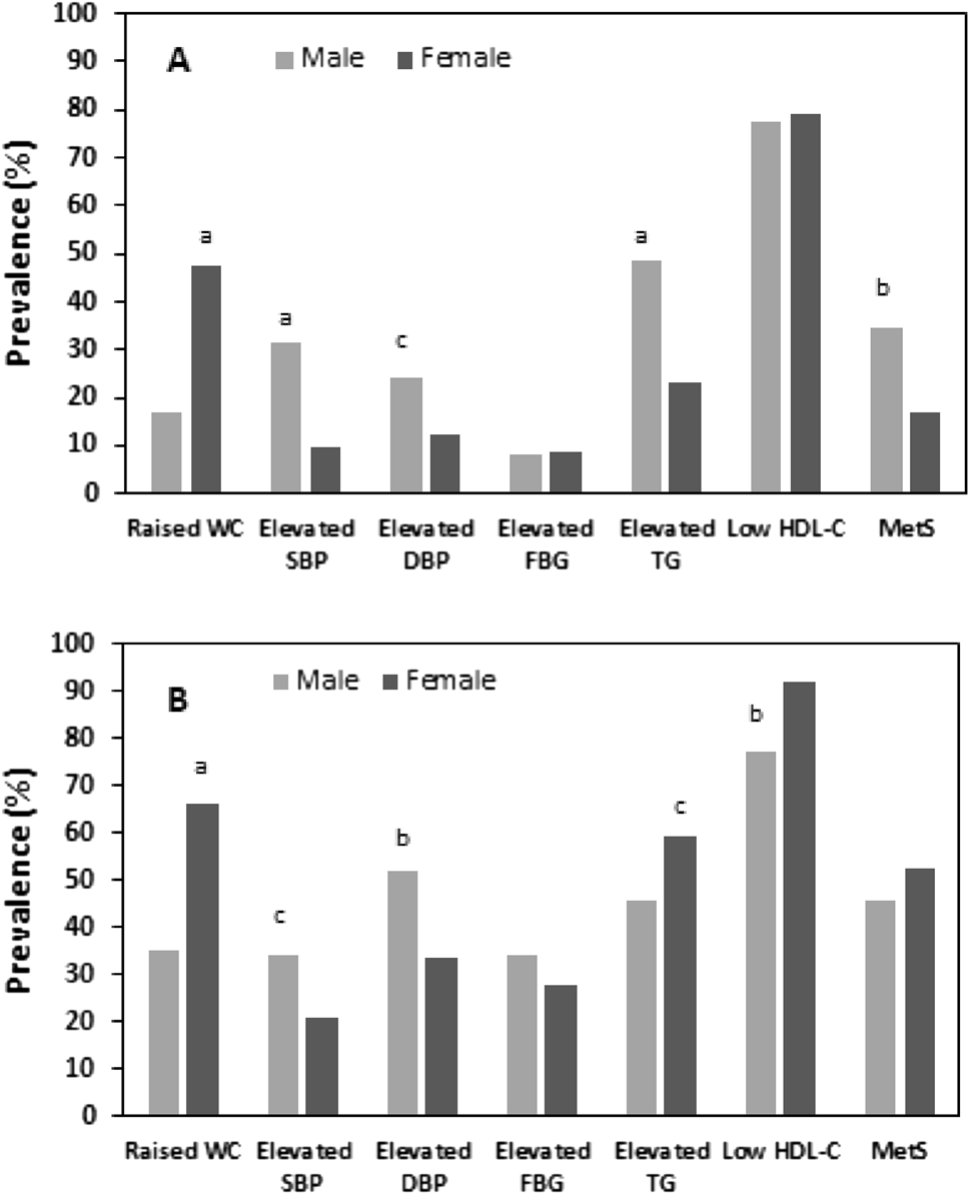
Prevalence of MetS and its components between the gender groups in students (**A**) and staff (**B**). ^a^P < 0.001, ^b^P < 0.001 and ^c^P < 0.05 when compared between male–female groups. P-values are derived from Chi-square test.

MetS was prevalent in 27.7% of students and 47.7% of academic staff. Among students, the prevalence of MetS was significantly higher in males (34.8%) than females (17.2%) (p < 0.01) (Table 2). On the other hand, the prevalence of MetS among staff was higher in female staff (52.3%) than male staff (45.8%), although the difference was not statistically significant. There were also significant gender-based differences in the prevalence of MetS components among students and staff (Fig. 1). The prevalence of MetS was found to be 16.8%, 84.6%, and 100% among healthy, hypertensive, and diabetic students, respectively. Similarly, among staff, the prevalence of MetS was 29%, 56.8%, and 56.5% in the healthy, hypertensive, and diabetic groups, respectively. The prevalence of MetS was higher in individuals with hypertension and diabetes among students (100%) and staff (93%).

**Table 2:** Prevalence of MetS in different groups.

	N			MetS (%)			
	Overall	Male	Female	Overall	Male	Female	P-value
Students							
Healthy-control	239	135	104	16.8	21.6	10.6	0.030
Hypertensive	33	29	4	84.6	87.0	66.7	0.408
Diabetic	3	1	2	100.00	100.0	100.0	–
Hypertensive-diabetic	6	3	3	100.00	100.0	100.0	–
Overall	281	168	113	27.7	34.8	17.2	0.002
Staff							
Healthy-control	155	103	52	29.0	23.3	40.4	0.022
Hypertensive	81	66	15	56.8	57.6	53.3	0.492
Diabetic	23	17	6	56.5	58.8	50.0	0.537
Hypertensive-diabetic	43	30	13	93.0	90.0	100.0	0.329
Overall	302	216	86	47.7	45.8	52.3	0.186

Hypertensive: participants who had hypertension but no diabetes, Diabetic: participants who had diabetes but no hypertension, Hypertensive-diabetic: participants who had both hypertension and diabetes. P-values are derived from differences in MetS% between males and females. P-values are derived from Chi-square test.

### Prevalence of MetS and its components by age groups

The highest prevalence of MetS was observed in the >23 years age group in students (45.2%) and > 50 years age group in staff (55.8%) (Table 3). An increased trend was found in the prevalence of MetS and its major components with age in both student and staff groups. Both genders in students and staff groups had the highest WC and TG levels in the increased age group. The most common component of MetS was low levels of HDL-C, affecting 78% and 81.4% of the sample in students and staff groups, respectively.

**Table 3:** Prevalence (%) of MetS and its components in different age groups.

	Parameters	Age groups					P-value
	Age (years)	Overall	≤ 19	20–21	22–23	> 23	
Students	MetS	27.7	4.9	18.2	39.2	45.2	0.000
	Raised WC	25.5	16.7	27.6	16.3	32.8	0.242
	Elevated SBP	22.9	12.5	19.5	29.2	29.0	0.148
	Elevated DBP	19.4	7.5	16.9	31.3	21.0	0.039
	Elevated FBG	8.2	0.0	9.1	11.8	9.7	0.189
	Elevated TG	39.0	9.4	21.7	45.1	66.1	0.000
	Low HDL-C	78.0	59.4	65.0	88.2	91.9	0.000

	Parameters	Age groups					P-value
	Age (years)	Overall	≤ 30	31–40	41–50	> 50	
Staff	MetS	47.7	37.5	43.4	54.6	55.8	0.105
	Raised WC	47.9	47.1	45.3	43.8	59.1	0.772
	Elevated SBP	30.3	14.8	23.6	433	37.2	0.001
	Elevated DBP	46.7	16.7	41.5	69.1	46.5	0.000
	Elevated FBG	32.5	12.5	31.1	37.1	51.2	0.000
	Elevated TG	49.7	50.9	51.4	45.2	54.1	0.752
	Low HDL-C	81.4	78.2	79.0	82.8	89.5	0.472

P-values are obtained Chi-square test.

### Regression analysis to determine factors associated with MetS

Based on multivariate logistic models (Tables 4 and 5), the important risk factor for MetS in both genders was age, with students being at risk if they were 22 years (OR 10.42, CI 2.23–48.72) or older (OR 13.49, CI 2.96–61.55), while staff were at risk if they were 40 years (OR 2.20, CI 1.11–4.36) or older (OR 2.33, CI 1.10–5.32) (at least p < 0.05 for all cases). Additionally, a higher BMI was found to be significantly associated with a higher risk of MetS in both students (OR 9.78, CI 2.64–36.27) and staff (OR 4.92, CI 2.36–10.22) groups (at least p < 0.01 for all cases). Among students, those with hypertension (OR 33.02, CI 8.38–130.19) and dyslipidemia (OR 21.92, CI 2.92–164.47) and who were physically less active (OR 4.45, CI 1.12–17.76) or smoked (OR 4.31, CI 1.10–16.94) were more likely to develop MetS (at least p < 0.05 for all cases). On the other hand, staff who had hypertension (OR 1.70, CI 1.01–2.86), were both hypertensive and diabetic (OR 18.70, CI 5.60–62.42), or had dyslipidemia (OR 12.71, CI 3.76–43.01) were found to be at an increased risk of MetS (at least p < 0.05 in all cases). Gender did not seem to have a significant association with the risk of developing MetS in both groups.

**Table 4:** Evaluation of risk factors associated with MetS in students by multivariate logistic regression analysis.

Risk factors	OR	95% CI	P-value
Gender			
Female	Reference		
Male	0.52	0.26–1.02	0.059
Age			
≤ 19	Reference		
20–21	3.84	0.82–17.96	0.088
22–23	10.42	2.23–48.72	0.003
> 23	13.49	2.96–61.55	0.001
BMI			
Normal	Reference		
Overweight	3.64	1.81–7.29	0.000
Obesity	9.78	2.64–36.27	0.001
Hypertension			
No	Reference		
Yes	33.02	8.38–130.19	0.000
Diabetes			
No	Reference		
Yes	1.12	0.40–2.20	0.675
Hypertensive-diabetic			
No	Reference		
Yes	1.05	0.50–1.80	0.770
Dyslipidemia			
No	Reference		
Yes	21.92	2.92–164.47	0.003
Physical activity			
Adequate/high	Reference		
Low	4.45	1.12–17.76	0.039
Smoking			
No	Reference		
Yes	4.31	1.10–16.94	0.037

Multivariate logistic regression was applied to evaluate the relationship between MetS and associated factors. Values are presented as OR (95% CI). *OR* odds ratio, *CI* confidence interval.

**Table 5:** Evaluation of risk factors associated with MetS in staff by multivariate logistic regression analysis.

Risk factors	OR	95% CI	P-value
Gender			
Male	Reference		
Female	1.52	0.90–2.56	0.116
Age			
≤ 30	Reference		
31–40	1.30	0.67–2.53	0.441
40–50	2.20	1.11–4.36	0.025
> 50	2.33	1.10–5.32	0.044
BMI			
Normal	Reference		
Overweight	1.67	0.88–3.17	0.115
Obesity	4.92	2.36–10.22	0.000
Hypertension			
No	Reference		
Yes	1.70	1.01–2.86	0.046
Diabetes			
No	Reference		
Yes	1.48	0.63–3.50	0.370
Hypertensive-diabetic			
No	Reference		
Yes	18.70	5.60–62.42	0.000
Dyslipidemia			
No	Reference		
Yes	12.71	3.76–43.01	0.000
Physical activity			
Adequate/moderate	Reference		
Low	0.84	0.43–1.65	0.612
Smoking			
No	Reference		
Yes	1.86	0.87–4.00	0.110

Multivariate logistic regression was applied to evaluate the relationship between MetS and associated factors. Values are presented as OR (95% CI). *OR* odds ratio, *CI* confidence interval.

## Discussion

This study aimed to determine the prevalence of MetS among university students and academic staff in Bangladesh and identify potential risk factors. So far, this is the first study that estimated the prevalence of MetS in student and staff populations within the university context of the country. Our study found that 27.7% of students and 47.7% of staff had MetS. A significant difference in MetS prevalence was observed between male and female students. In both student and staff groups, an increasing trend in MetS and its components was observed with age. The most common component of MetS was low levels of HDL-C, impacting 78% of students and 81.4% of staff. Risk factors associated with MetS in students included age, BMI, hypertension, dyslipidemia, low physical activity, and smoking. For academic staff, the significant factors were age, BMI, hypertension, and dyslipidemia.

In this study, it was found that the prevalence of MetS is lower among students but higher among staff members compared to the estimates reported by Chowdhury et al., where MetS was prevalent in 37% of the Bangladeshi population^11^. On the other hand, in a separate study by Gupta et al., MetS was found in only 15.2% of Bangladeshi adults^17^. The difference in results could be attributed to the fact that our study focused on a specific population within a university context, while Chowdhury et al. and Gupta et al. conducted studies at the local/community level and included participants from both rural and urban areas^11,17^.

Our findings from the student group can be compared to other studies conducted in different countries. For example, Malik et al. conducted a study in Pakistan and reported a lower prevalence of MetS among medical college students, with 6.48% in males and 1.13% in females^31^. In another study of US college students, the estimated prevalence of MetS was 12% in males and 6% in females^32^. A study conducted at West Virginia University in the USA found the prevalence of MetS to be 15% (18.8% in males and 11.1% in females) among their students^33^. A systematic review by Ampo-Arias et al. estimated the prevalence of MetS among university students in America, Asia, and Europe, which included 16 studies conducted between 2000 and 2016, using NCEP-ATP III criteria^34^. The review showed that the prevalence of MetS among students was up to 19.2%^34^. However, a recent study conducted in Iraq reported a higher prevalence of MetS (41.3%) among university students, with a higher frequency among female students (66.9%)^35^. Our finding is consistent with other studies that show a higher prevalence of MetS in male students than in female students^31,33^.

The prevalence of MetS in our staff group is higher than in a study conducted in India. The study reported a prevalence of 20.5% among teaching staff of an Engineering college, with a higher prevalence in females (25.32%) than in males (19.31%)^36^. On the other hand, a low prevalence of MetS was found among University staff in Iran, where MetS was prevalent in 13.1% of the participants, with a higher prevalence in females than males using NCEP-ATP III guidelines^37^. Similar to our study, the prevalence of MetS was higher in females than in males in both studies. However, another study at a Malaysian University reported the prevalence of MetS at 20.6%, with a higher prevalence in males (24.9%) than in females (18.3%)^38^.

In our study, BMI was strongly correlated with MetS among the students, consistent with other studies that showed a significant relationship between BMI and MetS^31,33^. It has been demonstrated that BMI is associated with an increased risk of developing MetS in adulthood^39^. In our staff group, increased BMI was significantly associated with MetS. Similar findings were reported in other studies, where BMI caused an increased risk of developing MetS among academic staff^36,38^. In obese individuals, breakdown of adipose cells releases free fatty acids and cytokines, such as tumor necrosis factor-alpha (TNF-α). These substances hinder the signal transduction pathways that rely on phosphatidylinositide-3-kinase, which, in turn, reduces glucose uptake in the liver and skeletal muscles^3,40^. Consequently, the pancreas is overburdened to secrete more insulin, leading to hyperglycemia or diabetes^41^.

In the present study, hypertension and dyslipidemia were significantly associated with MetS in student and staff groups. Of the MetS components, low HDL-C was the most prevalent form in students and staff. Similar findings were reported in other studies where hypertension and dyslipidemia were the independent risk factors of MetS^31,38^, suggesting these groups are at increased risk of developing cardiovascular disease. We also observed that age was significantly associated with MetS in students and staff groups, which is supported by other study findings, where increased age has been shown as an independent risk factor of MetS^11,17,38^. It is assumed that over time, the blood vessels tend to lose their elasticity and become more resistant, which can hinder the flow of blood. This, in turn, can result in poor circulation, leading to the accumulation of lipids in the abdomen and the release of free fatty acids into the bloodstream. These changes can cause an increase in levels of insulin resistance and serum triglycerides^42^.

Furthermore, inadequate physical activity and smoking significantly contributed to MetS, especially in students. Sedentary work and sitting tasks are more common in teaching than in general workers. Although physical activity did not significantly affect MetS among staff, sedentary behaviours may increase the risk of developing MetS^43,44^. In the student group, smoking showed a significant association with MetS. These findings were similar to previous studies^45–48^. However, no association was found in other studies^49^.

Regarding gender difference, the prevalence of MetS was about two times higher among male students than female students. This could be a reason hypertension prevalence was significantly higher among male students (16.9%) than female students (3.3%), which may influence the MetS rate in male students. In addition, male students might be less aware of food and body weight maintenance than female students. On the other hand, MetS prevalence was higher in female staff than in the male staff; even the prevalence of hypertension was higher in male staff. This might be related to some factors; for example, the physiological and metabolic changes that occur during menopause are a result of a decrease in estrogen levels, which impact lipid metabolism, energy consumption, insulin resistance, and body fat composition^50,51^. This hormonal shift causes a transition from a gynecoid body shape to an android one with an increase in abdominal and visceral fat accumulation, leading to a higher risk of cardiovascular disease and metabolic issues^50,51^. Furthermore, pregnant women are more likely to be obese, and multiple pregnancies can further increase the risk of developing MetS^52^. Overall, our findings indicate a widespread prevalence of MetS among students and academic staff in the University context in Bangladesh. These data identify the importance of implementing health programs to screen for MetS components and prevent the onset of chronic diseases among the university population. It is imperative for University administrators across the country to take action and prioritize the health of their students and staff. Although sex and age are non-modifiable risk factors for MetS, modifiable lifestyle behaviors such as sleep patterns, dietary habits, physical activity levels, and stress management practices may offer more significant interpretations.

One of the key strengths of the study was its use of a precise case definition that relied on blood tests to confirm diagnoses of dyslipidemia and diabetes. Additionally, the study's analysis took into account numerous factors that could potentially increase the risk of MetS. Our study also had some limitations. Due to the study’s cross-sectional nature, it was impossible to establish a causal relationship between MetS and certain risk factors. Additionally, it is important to note that the sample size for this study was relatively small; therefore, more extensive studies are needed to better understand the prevalence of MetS among university students and staff across the country. Lastly, it is worth mentioning that the prevalence of MetS was determined based on a single measurement of blood parameters, which may have resulted in minor inaccuracies.

## Conclusion

A considerably higher prevalence of MetS was observed among students and academic staff. Older individuals showed a higher likelihood of having MetS. For students, significant factors associated with MetS were age, BMI, hypertension, dyslipidemia, low-intensity physical activity, and smoking. Academic staff showed significant associations with age, BMI, hypertension, and dyslipidemia. Interventions aimed at improving lifestyle behaviours and identifying high-risk individuals may help reduce the burden of MetS. These findings emphasize the importance of implementing health screening and interventions for students and staff in various university settings in Bangladesh. The data from such screenings can inform researchers, public health officials, and university administrators to implement effective interventions to prevent chronic disease progression in academic settings. Health promotion activities, such as healthy diet and exercise programs, should be implemented more rigorously. Conducting future research with more representative samples can provide precise insights into the prevalence of MetS among different subgroups of the university population, which can help design and implement interventions for high-risk groups.

## Data Availability

The datasets used and/or analysed during the current study are available from the corresponding author upon reasonable request.
